# Liquid permeation and chemical stability of anodic alumina membranes

**DOI:** 10.3762/bjnano.8.60

**Published:** 2017-03-06

**Authors:** Dmitrii I Petukhov, Dmitrii A Buldakov, Alexey A Tishkin, Alexey V Lukashin, Andrei A Eliseev

**Affiliations:** 1 Department of Chemistry, Lomonosov Moscow State University, Moscow 119991 Leninskie hills 1–3, Russia; 2 Department of Materials Science, Lomonosov Moscow State University, Moscow 119991 Leninskie hills, Russia

**Keywords:** anodic alumina, carbon-modified membranes, membrane stability, microfiltration membranes, ultrafiltration membranes

## Abstract

A study on the chemical stability of anodic alumina membranes and their performance in long-term water and organic solvent permeation experiments is reported. Anodic alumina possesses high stability for both protonic and aprotonic organic solvents. However, serious degradation of the membrane occurs in pure water, leading to a drastic decrease of permeance (over 20% of the initial value after the passing of 0.250 m^3^/m^2^ of pure water). The drying of the membrane induces further permeance drop-off. The rate of membrane degradation strongly depends on the pH of the penetrant solution and increases in basic media. According to ^27^Al NMR and thermogravimetry results, the degradation of the membranes is associated with the dissolution of water-soluble [Al_13_O_4_(OH)_24_(H_2_O)_12_]^7+^ polyhydroxocomplexes and their further redeposition in the form of [Al(OH)_4_]^−^, resulting in channels blocking. This process intensifies in basic pH due to the high positive charge of the anodic alumina surface. An approach for improving anodic aluminum oxide stability towards dissolution in water by carbon CVD coating of the membrane walls is suggested.

## Introduction

Porous anodic aluminum oxide (AAO), developed as a protective coating, has received close attention of the membrane community due to its unusual porous structure represented by piercing cylindrical channels and exceptional transport characteristics. A very narrow pore size distribution, low pore tortuosity and controllable membrane porosity make AAO one of the top performers given its permeability/pore diameter ratio [1]. The synthetic procedure of AAO membranes enables significant tunability of the various porosity parameters (channel diameter 5–400 nm, interpore distance 20–600 nm, porosity 10–50%, thickness from 500 nm to 300 µm) [2–4]. It also allows membranes with a hierarchical porous structure to be obtained, where a supporting macroporous layer (aimed to provide mechanical durability) merges into the microporous selective skin layer [5]. All these issues make AAO extremely attractive for baromembrane processes, including micro- and ultrafiltration, pervaporation, emulsification and membrane catalysis. AAO membranes are abundantly used as solid porous supports for asymmetric gas separation membranes [6–7] and have been tested in industrial applications as porous condensing layers for on-site associated gas conditioning [8–9]. However, most of AAO membrane functions are associated with gas separation processes [3,5,7–8,10–12], while only few reports can be found for liquid media [1,13–15]. In contrast, the narrow pore size distribution of anodic alumina membranes should result in a sharp cut-off curve and excellent filtration characteristics [1]. The possible reason for low publishing and application activity in the field is the degradation of AAO membrane materials, resulting in a substantial decrease of the solvent flux through the membrane with time [15].

Therefore, in the present study, we focused on the chemical stability of the membrane and the stability of the membrane performance towards water and organic solvents, as well as analysis of the AAO microstructure during degradation of the membranes. Several pathways for improving the AAO stability and performance are suggested.

## Results and Discussions

**Figure 1:**
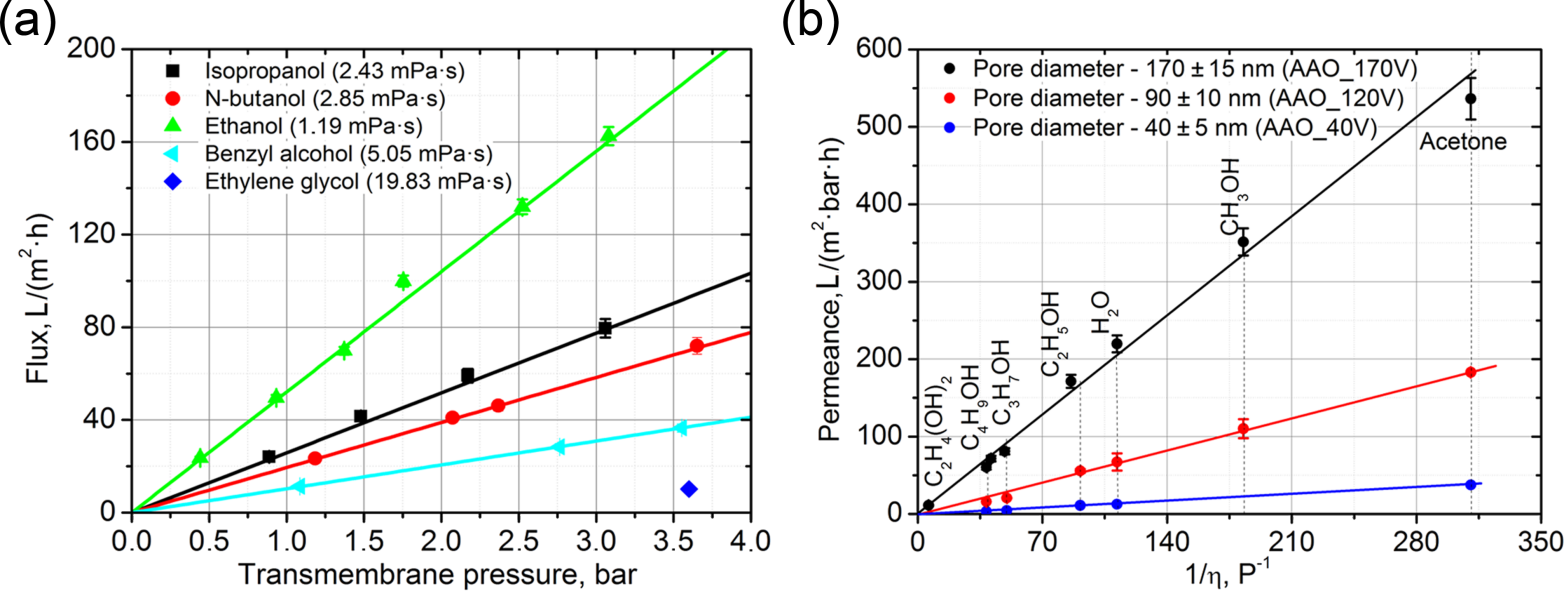
(a) Dependence of liquid flux vs transmembrane pressure for membrane AAO_120V with average pore diameter of 90 ± 10 nm and thickness of 100 μm and (b) dependence of liquid permeance on the inverse viscosity of the liquid for 100 μm membranes AAO_40V (*D*_pore_ = 40 nm; 10% porosity), AAO_120V (*D*_pore_ = 90 nm; 10% porosity) and AAO_170V (*D*_pore_ = 170 nm; 22% porosity).

The permeation of liquids through freshly prepared AAO membranes agrees well with Darcy’s law: liquid flux linearly depends on transmembrane pressure (Figure 1a) and the slope of the curve is inversely proportional to the viscosity of the media (Figure 1b). In concert with a quadratic increase of the permeance with increasing membrane pore diameter (Figure 1b), it clearly reveals a Poiseuille flow mechanism for liquid transfer through AAO nanochannels. Notably, the water permeability of a 100 µm thick AAO membrane with a pore diameters of 170 nm attains 220 L/(m^2^·bar·h), which substantially exceeds that of most porous filters having the pore same size and is nearly equal to the permeability of track-etched membranes [16].

**Figure 2:**
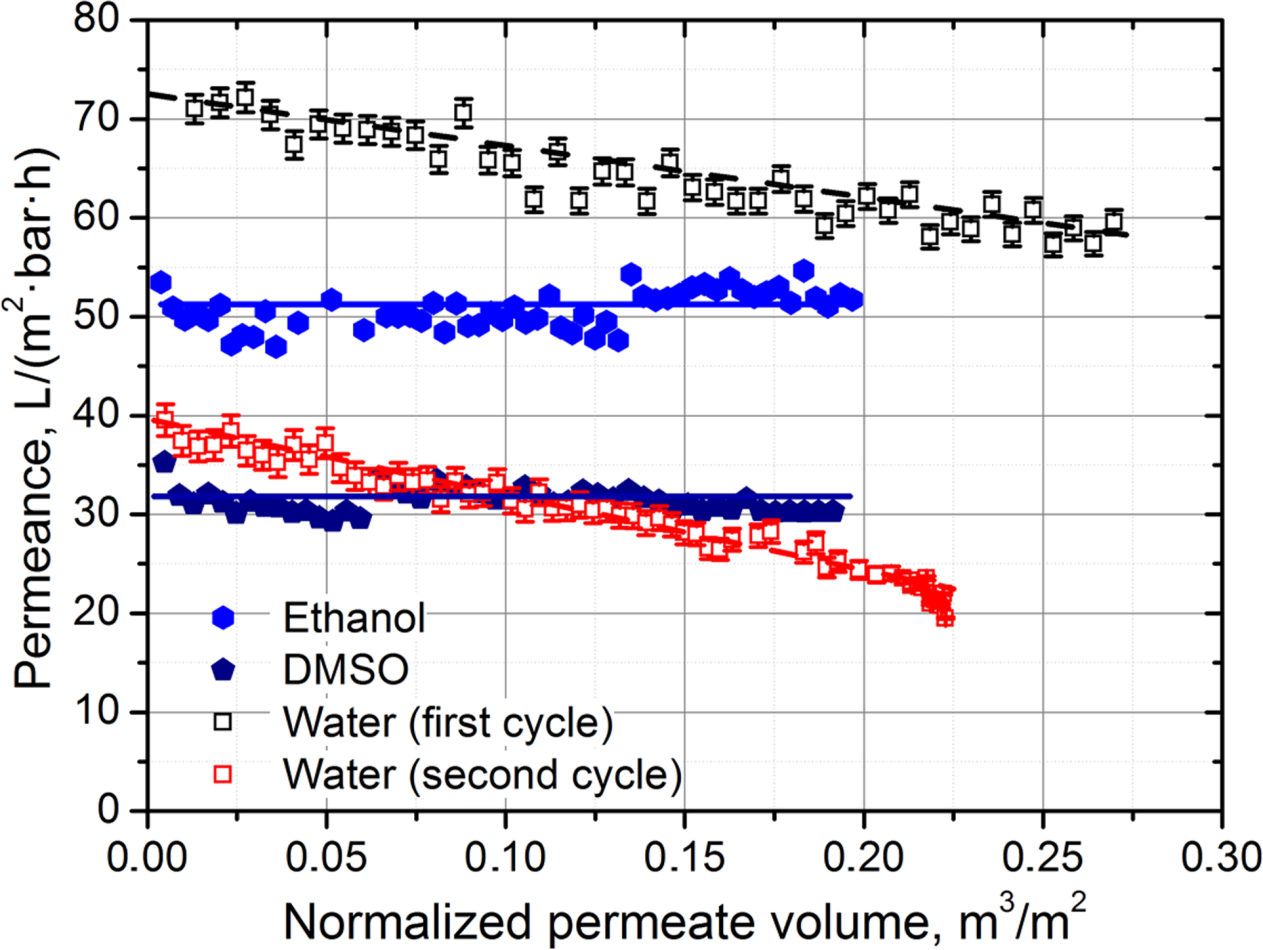
Dependence of the AAO_120V membrane (*D*_pore_ = 90 nm, 10% porosity, thickness – 100 μm) permeance for DMSO, ethanol and water on the specific permeate volume in long-term permeation experiments.

However, pure water flux through the AAO membrane was found to decrease with time, indicating degradation of the membrane material, which persuaded us to conduct long-term permeability experiments. The long-term flux stability was studied for both protonic and aprotonic solvents with different viscosity. The dependence of membrane permeance for different solvents on a specific permeate volume (per unit area) is represented in Figure 2. The parameters of the experiments are also summarized in Table 1. It can be seen that the permeance for both protonic and aprotonic organic solvents is quite stable during the long-term measurement, with small alterations explained by the slight temperature variation during experiments. In contrast, in the case of water filtration, the flux decreases substantially with an initial slope of −53.2 ± 3.2 bar^−1^·h^−1^. Moreover, a drastic decrease of the AAO membrane water permeance from 73 ± 3 L/(m^2^·bar·h) to 58 ± 1 L/(m^2^·bar·h) was observed after drying the membrane for 12 h at room temperature, followed by second-cycle measurements of water permeance. In the second cycle, the permeance also decreased with nearly the same slope (Figure 2, Table 1). The total permeance loss during two cycles (500 L/m^2^ total throughput) equaled 72% of the initial value.

**Table 1:** Long-term permeation parameters for the AAO_120V membrane with an average pore diameter of 90 ± 10 nm, thickness 100 μm and porosity 10%.

Solvent	Viscosity, mPa·s	Initial permeance, L/(m^2^·bar·h)	Solution pH	Permeability slope, bar^−1^·h^−1^	Membrane weight loss, %	Al concentration in permeate, mg/L

DMSO	2.02	31.5 ± 0.3	–	−2.2 ± 2.8	0	−
ethanol	1.19	49.0 ± 0.5	–	16.9 ± 3.8	0	−
water	0.89	73 ± 3	3	20.5 ± 2.2	0.013	4.4
			4.5	8.4 ± 0.4	0	2.8
			6 (first cycle)	−53.2 ± 3.2	0	0.04
			6 (second cycle)	−69.6 ± 6.9	0	n.a.
			6.5	−366 ± 16	0	2.1
			7.6	−800 ± 30	0	3.2
			9	−1980 ± 120	0	6.7

**Figure 3:**
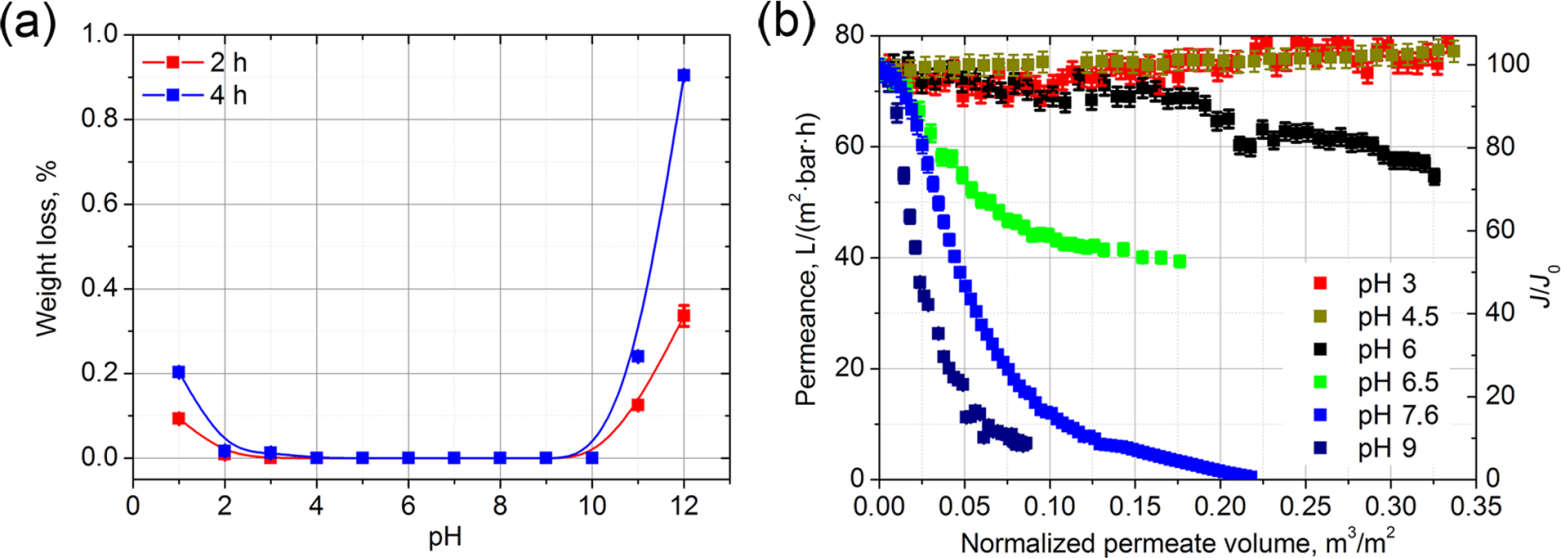
(a) Chemical dissolution of AAO at different pH and (b) dependence of AAO membrane permeance with pH during flow of buffered solutions.

Notably, among a large number of studies concerning water permeance through AAO membranes [1,13–15], there has been only one reported case of the decrease of the liquid flux during filtration [15]. As only high purity distilled and deionized water was used in experiments, we suggested that the degradation of the membrane occurred by dissolution of the anodic aluminum oxide and its further redeposition on the pore walls. For a detailed determination of the anodic alumina degradation mechanism, chemical dissolution and buffered solution permeation experiments were performed for the AAO_120V membrane (Figure 3a,b). AAO membranes were found chemically stable over a wide pH range (3–9) with a mass loss during two hours with solvent immersion below the accuracy limit (0.01%, Table 1). The obtained stability range slightly exceeds the values reported by Mardilovich et al. (pH 5.0–8.2) for the membranes obtained by anodization at 40 V [17]. Alterations of the pH stability ranges might be explained by the difference in chemical composition of membranes obtained in “mild” and “hard” anodization conditions. The membranes obtained at high voltages are known to contain nearly the same quantity of absorbed anions, but a much lower amount of the absorbed water [18] (see also Supporting Information File 1, Figures S1, S2 and Table 2). The greatest differences in chemical composition are presumed to localize in the outer anodic oxide layer [4].

**Table 2:** Results of fitting the ^27^Al NMR spectra for membranes obtained in mild (AAO_40V) and hard (AAO_120V) anodization conditions in the initial state (i) and after pure water filtration (w).

Sample	Pore diameter, nm	Water permeance L/(m^2^·bar·h)	Peak area, %				Water loss below 500 °C, %	CO_2_ loss at crystallization, %

			Al(4), (≈60 ppm)	Al(5), (≈35 ppm)	Al(6), (≈0 ppm)	Al(6), (≈7 ppm)		

AAO_40V_i	40 ± 5	12.4 ± 0.5	19.5 ± 0.1	50.0 ± 0.2	27.1 ± 0.1	3.4 ± 0.1	3.7	5.3
AAO_40V_w		7.0 ± 0.4	20.1 ± 0.1	51.8 ± 0.2	28.1 ± 0.1	–	4.3	6
AAO_120V_i	90 ± 10	73 ± 3	17.1 ± 0.3	53.0 ± 0.6	29.9 ± 0.5	0.5 ± 0.1	0.3	6
AAO_120V_w		46 ± 1				–	1.2	6.3

Contrary to the dissolution tests, the buffered solution permeation experiments indicated significant changes in permeance during long-term experiments within the chemical stability window. In case of the permeation of the solution with pH 3 and 4.5, a slight increase of the permeance was observed, indicating a partial dissolution of the membrane with an increase of the effective pore diameter. This was proved by the presence of 4.4 and 2.8 mg/L of Al^3+^ ions in permeate that for a buffer solution of pH 3 and 4.5, respectively (Table 1). For solutions with neutral and basic pH, a decrease of the permeance occurred. The permeate solution also contained dissolved aluminum (Table 1).

**Figure 4:**
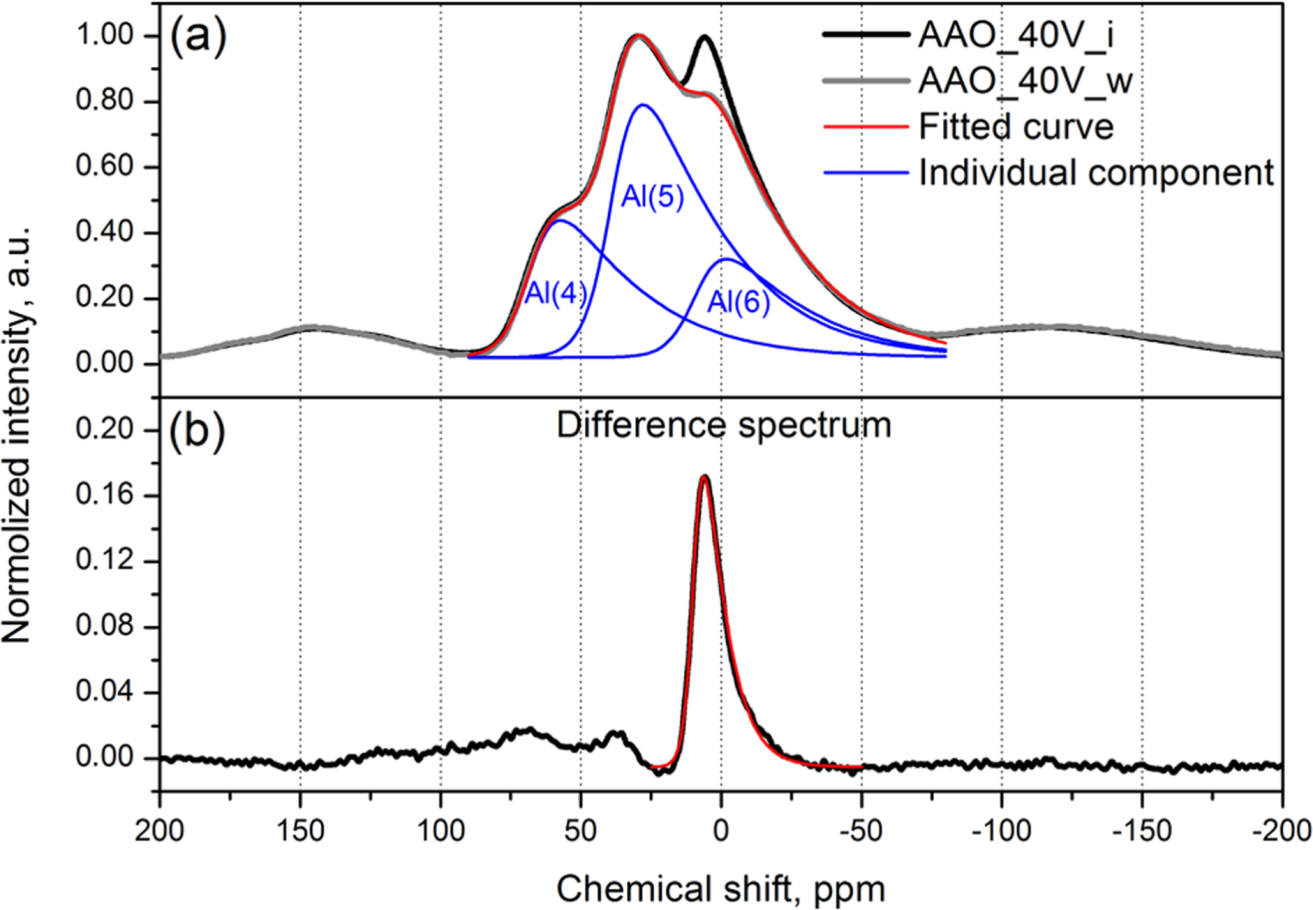
^27^Al solid-state NMR spectra of the initial membrane with pore diameter 40 ± 5 nm (AAO_40V_i) and the same membrane after 500 L/m^2^ pure water filtration (AAO_40V_w) (a) and the difference spectrum (b).

Alumina dissolution from the pore walls was also proved by ^27^Al solid state NMR spectroscopy of initial membranes and membranes after 500 L/m^2^ pure water filtration. The spectra for initial membrane AAO_40V was obtained by mild anodization and this membrane after water filtration illustrates very close profiles with the only difference represented by a peak at ≈7 ppm (Figure 4). The difference spectrum (Figure 4b) is fitted well by a single component with an asymmetric resonance signal shape centered at 7.5 ppm. The remaining alumina signals for both the initial membrane and that after water filtration are fitted well by the combination of the asymmetric resonance signals of tetra-, penta- and hexa-coordinated alumina, assuming the same half-widths and asymmetry parameters for different atoms. The fitting results are represented in Table 2.

Notably, the width of an additional component persistent in the initial membrane is much lower as compared to other components in the spectrum (Figure 4), which indicates much higher mobility for this atom. This peak corresponds well to octahedrally coordinated aluminum in polymerized [Al_13_O_4_(OH)_24_(H_2_O)_12_]^7+^ polyhydroxocomplexes or alumina atoms partially coordinated by C_2_O_4_^2−^ [19]. The first hypothesis seems more plausible based on thermogravimetry results presented in Supporting Information File 1 (Figure S1). The intensity of the additional component in the NMR spectra is much lower in the case of the AAO_120V membrane (pore diameter 90 ± 10 nm) obtained by hard anodization, which corresponds with the quantity of weakly bounded water (see Supporting Information File 1, Figure S1). Thus ^27^Al NMR indicates the removal of aluminum atoms partially coordinated by oxalic acid or water-soluble aluminum polyhydroxocomplexes during water filtration experiments. This also suggests the initial location of those complexes at the surface of the outer anodic oxide layer. Despite the fact that several papers report on the formation of an AAO structure by coagulation of colloid alumina species [20–21], our results can unlikely be used as proof for the suggested mechanism because alumina polycation formation pH (≈5) strongly changes the pH of the electrolyte used during anodization (≈1).

However, dissolution of the membrane material does not explain the loss of membrane permeability in the long-term permeation experiments. On the other hand, since the polycondensation reaction for alumina hydroxocomplexes deposited on AAO walls can be reversible before oxolation takes place, both dissolution and deposition processes appear all over the film thickness. The efficiency of both reactions is dictated by the equilibrium constants. While at the entrance of the membrane equilibrium is strongly shifted to the dissolution of the alumina containing species, at the exit of the membrane, both dissolution and deposition reactions have the same rates. This leads to an effective transport of water-soluble alumina species to the permeate side of membrane during water filtration, encouraging possible blockage of channels. The effect is strongly enhanced by a slight gradient of pH within the pore due to the neutralization reaction by dissolved aluminium oxide.

**Figure 5:**
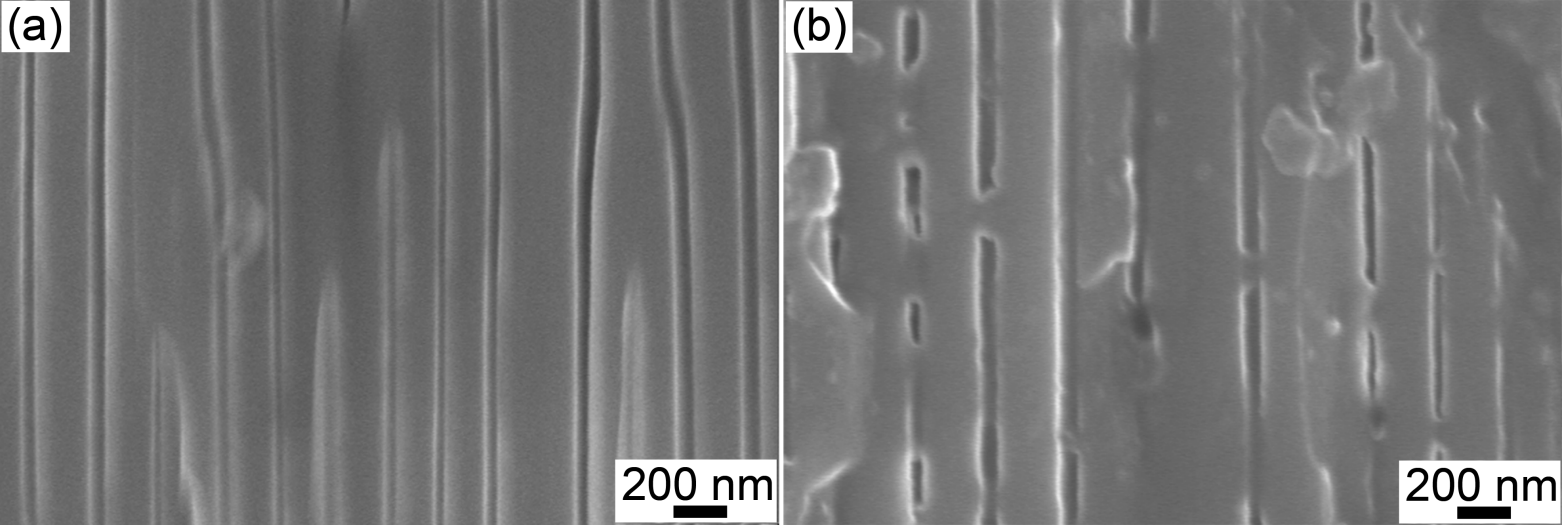
Scanning electron micrograph of the AAO_120V membrane cross section before (a) and after the filtration experiment (b).

The physical blockage of AAO nanochannels is well observed by scanning electron microscopy from cross sections of alumina membranes used in the permeability experiments with pH 9 buffered solution (Figure 5). Small necks were found to block the majority of the pores. Moreover, the chemical composition of the blocking material was noted to be the same as that of the pore walls. This explains the mechanism of the degradation of the membranes by dissolution of aluminum-containing species from the pore walls and its further redeposition.

**Figure 6:**
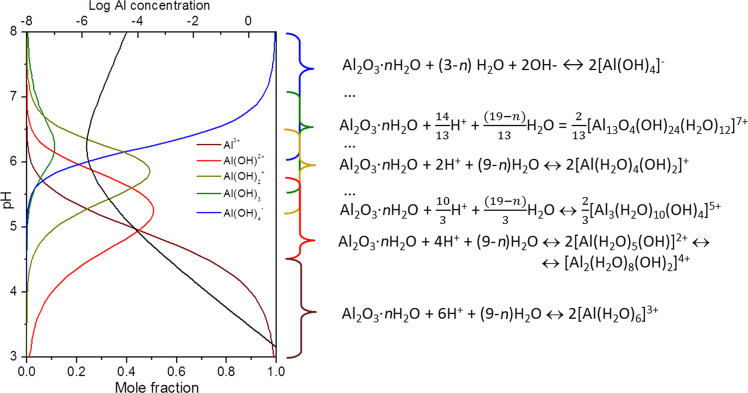
Concentration of the different aluminum complexes in solution depending on the pH of the media and dissolution reactions at different pH.

According to our model, the difference in the long-term permeability behavior of AAO membranes in acidic and basic media can obviously be attributed to the nature of the dissolved aluminum-containing species and their interactions with pore walls. Depending on the pH of the penetrating solution, one can assign a set of dissolution reactions for anodic alumina (Figure 6 [22]).

In an acidic or slightly acidic medium, the degradation involves dissolution of AAO with the formation of positively charged hydroxylated [Al(HO)(H_2_O)_3..5_]^2+^ and [Al(HO)_2_(H_2_O)_2..4_]^+^ alumina species which further can condense in polynucleic hydroxocomplexes.

These complexes are repelled from the AAO membrane surface, having an isoelectric point at pH 8.0 [23]. The charge of hydroxocomplexes that is dominant in water solution changes at pH ≈6. Taking into account a positive charge of the AAO surface at neutral pH values, this probably occurs in a solvent volume within the pore via hydrolysis reactions:


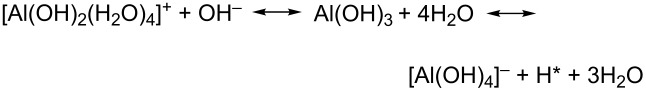


**Scheme 1:**
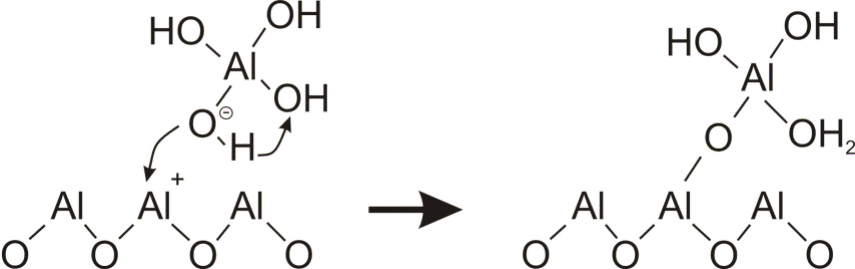
Polycondensation reaction.

**Scheme 2:**
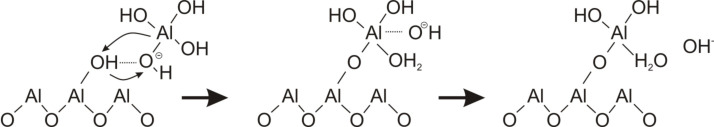
Condensation at Bronsted acid sites.

Despite the fact that Al(OH)_3_ can block the channels itself, this process can unlikely be considered favourable, especially at basic pH values, as an unbound aluminium hydroxide would be dissolved much more easily than the alumina of the pore walls. The condensation of the hydrolysis products fits with the experimental results much better, especially since negatively charged [Al(OH)_4_]^−^ would readily be attracted by the AAO surface. As [Al(OH)_4_]^−^ species can be detected in water solution in noticeable amounts starting from pH 5, the following reactions should be considered dominant for alumina redeposition leading to pore blocking in the pH range of 5–9 (Scheme 1). The condensation can also occur at Bronsted acid sites (Scheme 2).

Higher pH values lead to further dissolution of the AAO membrane (Figure 3b). Thus, the permeation of water solutions depends strongly on the pH of the medium. In acidic water solutions, the dissolution of alumina walls takes place; no redeposition of dissolved species occurs due to the high positive charge of the AAO surface. In the neutral pH range, below the isoelectric point of the AAO membrane, the further redeposition of dissolved [Al(OH)_4_]^−^ ensues in nanochannels. Moreover, as the efficiency of [Al(OH)_4_]^−^ generation in the solution grows exponentially with pH, the pore blockage rate also rapidly increases. At values greater than the isoelectric point, the redeposition efficiency decreases with increasing AAO negative charge; however, high pH values lead to the dissolution of the AAO material.

**Figure 7:**
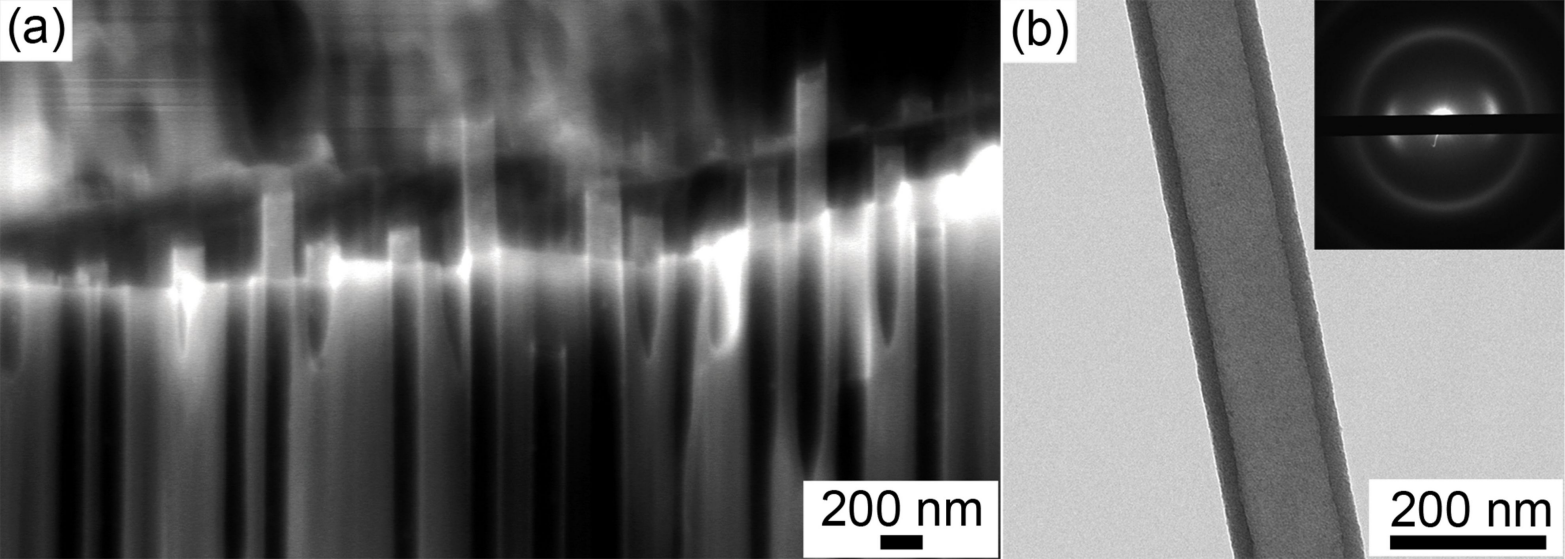
SEM (a) and TEM (b) micrographs of carbon nanotubes formed in anodic alumina membrane pores by CVD.

To avoid the permeability loss of anodic alumina in water solutions, and to take advantage of AAO membrane applications in aqueous medium, one needs to suppress the dissolution efficiency. The most convenient way involves oxolation and partial crystallization of hydroxides to crystalline alumina phases having extremely low solubility products. Unfortunately, thermal treatment of anodic alumina films is known to be accompanied by increasing brittleness and possible membrane curling [24–25]. However, none of the pure chemical methods (phosphate or chromate treatment) results in the necessary stability improvement towards dissolution. Therefore, we have tested the long-term water permeation stability of thermally treated AAO membranes. The temperature of 600 °C was chosen to induce the crystallization of AAO and to avoid permeance loss due to destruction of the porous structure. However, thermally treated membranes have also illustrated water permeation loss during the operation and after membrane drying between the cycles. To provide an additional protection, carbon CVD (by the pyrolysis of hydrocarbons) was also adopted in the same thermal treatment regime to provide a continuous 10 nm thick coating of the internal porous oxide surface (Figure 7). The details on the coating procedure and analysis of the composite membranes are given in Supporting Information File 1.

**Figure 8:**
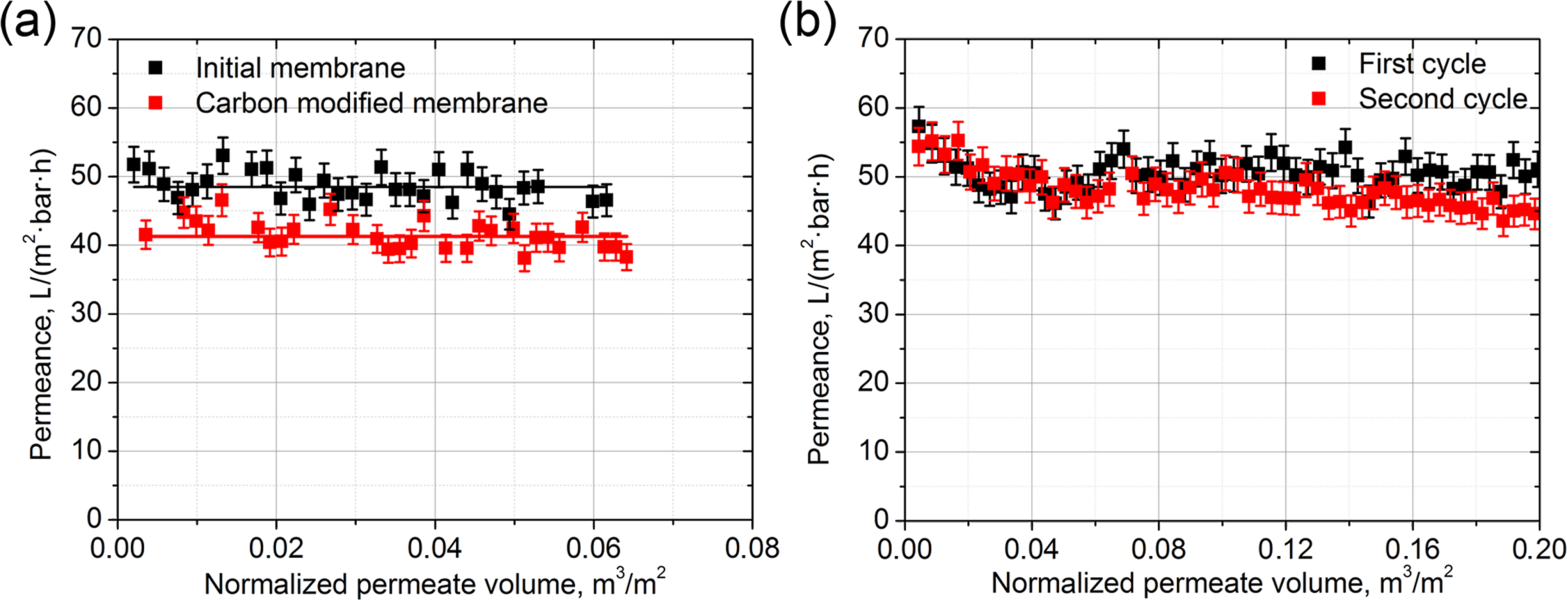
Comparison of ethanol permeance for initial membrane and the membrane modified with carbon (a) and the water permeance of the CVD-modified membrane (b).

The long-term permeability studies were carried out for pure AAO_120V and composite AAO_120V membranes modified with carbon coating (Figure 8). A slight decrease of the ethanol permeance from 49.4 ± 2.1 to 41.9 ± 0.5 L/(m^2^·bar·h) was observed for the composite membrane. In comparison with earlier published studies [26–27], we have not observed any improvement of the membrane performance due to slip flow. This can arise either from a decrease of the nanochannel diameter or from an insufficient smoothness of the carbon coating. In water permeation experiments, both the membranes illustrate a significant increase in membrane stability. Moreover, in both cases, the membrane permeance remains nearly the same after drying and the second utilization for water permeation. Only trace aluminum content was detected in permeate solutions. Despite this, we are unable to provide a quantitative comparison of long-term membrane performance for these two kinds of membranes as their permeability losses fall within measurement error limits; however, slightly better stability of carbon-coated membranes was noticed. Therefore, both the thermal treatment procedure and the CVD coating of the membrane with an amorphous carbon results in the stabilization of AAO membranes towards dissolution in water. This therefore allows for further utilization of AAO for baromembrane processing.

## Conclusion

In this study, the chemical stability of AAO membranes and the stability of the membrane performance towards water and organic solvents were reported. As-prepared anodic alumina membranes were found to possess high stability to both protonic and aprotonic organic solvents. However, significant degradation of the membrane performance was observed for water solutions due to dissolution of polymerized [Al_13_O_4_(OH)_24_(H_2_O)_12_]^7+^ polyhydroxocomplexes. This effect results in strong variations in the AAO membrane performance depending on the pH of the penetrant solution. In acidic or slightly acidic media, the dissolution of alumina leads to a slight increase of the membrane permeance. No redeposition of the dissolved species occurs due to the high positive charge of the AAO surface. In the neutral pH range, below the isoelectric point of the AAO membrane, further redeposition of dissolved [Al(OH)_4_]^−^ ensues in the nanochannels. Moreover, as the efficiency of [Al(OH)_4_]^−^ generation in the solution grows exponentially with pH, the pore blockage rate also rapidly increases. Beyond the isoelectric point, the redeposition efficiency decreases with increasing AAO negative charge; however, high pH values also lead to the dissolution of the AAO material. An approach for improving the AAO stability towards the dissolution in water by carbon CVD coating of the membrane walls was suggested. This method resulted in the stabilization of the AAO membranes, which could allow for the further utilization AAO for baromembrane processes.

## Experimental

### Sample preparation

Prior to anodization, aluminum foil was annealed and electro-polished according to procedure described elsewhere [28]. The anodization was carried out in a two-electrode cell. The electrolyte was pumped through the cell by a peristaltic pump, and its temperature was kept in the range of 0–2 °C during anodization. For the preparation of membranes with an average pore diameter of 40 nm and 90 nm, aluminium was anodized in 0.3 M H_2_C_2_O_4_ (98%, Aldrich) under mild and hard anodization conditions at 40 V and 120 V, respectively. A membrane with an average pore diameter of 170 nm was obtained by anodization in 0.1 M H_3_PO_4_ at 170 V. In the case of mild anodization conditions (40 V), a two-step anodization procedure was used for improving the membrane permeance. The 100 μm sacrificial layer formed by the first anodization was selectively removed in the solution containing 0.6 M H_3_PO_4_ + 0.2 M CrO_3_ at 60 °C. The second anodization was performed under the same conditions. In hard anodization conditions the voltage was increased to the target voltage of 120 V during the first step with a constant rate of 1 V·s^−1^ in order to avoid electrical breakdown of the oxide film on the first step. The thickness of the formed films was coulometrically controlled, resulting in 100 µm thick membranes [28].

After anodization, the oxide films were separated from the metallic substrates by selective metal dissolution in 0.5 M CuCl_2_ (5 vol % HCl). Subsequently, the pore bottoms were opened by chemical etching in 25 wt % H_3_PO_4_ aqueous solution at 25 °C followed by electrochemical detection of the pore opening [28].

To increase on the first step membrane stability, high-temperature dehydration was carried out by an annealing at 600 °C (2 h). To prevent curling or breaking of membrane during heat treatment, the heating and cooling rates were set to 1 °C/min. A protective carbon layer was deposited onto anodic alumina pore walls by CVD from 15 vol % C_3_H_6_ in He (150 mL/min) at 600 °C for two hours. The heating/cooling rates were also set to 1 °C/min.

### Sample characterization

Scanning electron microscopy (SEM) images were recorded on a Supra 50VP instrument (LEO) instrument. The deposited carbon layer was examined by transmission electron microscopy (TEM) after dissolution of the alumina matrix using a Zeiss Libra 200 FX instrument. Raman spectroscopy studies were carried out on a Renishaw InVia spectrometer using He–Ne laser excitation (633 nm). ^27^Al NMR spectra were recorded on a Bruker AVANCE II 400WB spectrometer at 104.3 MHz, using 0.7 ms (15°) pulses and 0.5 s recycle delays with magic-angle-spinning at 10 kHz. The chemical shifts of ^27^Al are given in ppm from external Al(H_2_O)_6_^3+^.

The liquid permeance measurements were performed using a dead-end Teflon cell at a temperature of 25 ± 2 °C. The permeability was calculated from the pressure difference measured by pressure transducers and the mass of accumulated permeate. The time step of the measurement was 5 min. Before the experiment, all tested liquids and solutions were prefiltered through Chromafil Xtra 0.2 μm filters. The chemical stability and buffer solution permeation experiments were performed using water solutions with different pH. For preparation solutions with pH in range from 3 to 9, the buffers listed in Table 3 were used. The composition of the buffered solutions was optimized to minimize the ionic strength difference and diminish the influence of the electrical double-layer thickness during permeation experiments.

**Table 3:** Composition of buffer solutions used in permeation experiments and chemical stability measurements.

pH	Buffer type	Concentration	

3	acetate	0.139 M CH_3_COOH	0.04 M CH_3_COONa
4.5		0.065 M CH_3_COOH	0.096 M CH_3_COONa
6	–	0.12 M NaCl	–
6.5	phosphate	0.026 M KH_2_PO_4_	0.033 M Na_2_HPO_4_
7.6		0.007 M KH_2_PO_4_	0.044 M Na_2_HPO_4_
9	carbonate-bicarbonate	0.09 M NaHCO_3_	0.01 M Na_2_CO_3_

## Supporting Information

Supporting Information File 1 contains the thermal analysis data of membranes obtained under “hard” and ”mild” anodization conditions, in addition to experimental details regarding the carbon coating and Raman spectrum of the obtained carbon coating.

File 1 Thermal analysis and membrane modification via CVD process.

## References

[1] Lee K P, Mattia D (2013). J Membr Sci.

[2] Li A P, Müller F, Birner A, Nielsch K, Gösele U (1998). J Appl Phys.

[3] de L. Lira H, Paterson R (2002). J Membr Sci.

[4] Lee W, Park S-J (2014). Chem Rev.

[5] Petukhov D I, Napolskii K S, Eliseev A A (2012). Nanotechnology.

[6] Chernova E, Petukhov D, Boytsova O, Alentiev A, Budd P, Yampolskii Y, Eliseev A (2016). Sci Rep.

[7] Inguanta R, Amodeo M, D'Agostino F, Volpe M, Piazza S, Sunseri C (2007). J Electrochem Soc.

[8] Petukhov D I, Surtaev V N, Eliseev A A (2015). Nauchno-technicheskiy vestnik OAO«NK «Rosneft».

[9] Pyatkov E S, Surtaev V N, Petukhov D I, Chernova E A, Lukashin A V, Solntsev K A, Eliseev A A (2016). Neftyanoe khozyaystvo - Oil Industry.

[10] Inada T, Uno N, Kato T, Iwamoto Y (2005). J Mater Res.

[11] Petukhov D I, Eliseev A A (2016). Nanotechnology.

[12] Petukhov D I, Berekchiian M V, Pyatkov E S, Solntsev K A, Eliseev A A (2016). J Phys Chem C.

[13] Lee K P, Leese H, Mattia D (2012). Nanoscale.

[14] Lee K P, Mattia D (2013). Ind Eng Chem Res.

[15] Itaya K, Sugawara S, Arai K, Saito S (1984). J Chem Eng Jpn.

[16] Mehta A, Zydney A L (2005). J Membr Sci.

[17] Mardilovich P P, Govyadinov A N, Mazurenko N I, Paterson R (1995). J Membr Sci.

[18] Lee W, Ji R, Gösele U, Nielsch K (2006). Nat Mater.

[19] Masion A, Thomas F, Tchoubar D, Bottero J Y, Tekely P (1994). Langmuir.

[20] Michelson C E (1968). J Electrochem Soc.

[21] Pashchanka M, Schneider J J (2011). J Mater Chem.

[22] Saukkoriipi J (2010). Theoretical study of the hydrolysis of aluminum complexes.

[23] Winkler B H, Baltus R E (2003). J Membr Sci.

[24] Wang D L, Ruan Y F, Zhang L C, Zhu W, Wang P F (2013). Cryst Res Technol.

[25] Choudhari K S, Sudheendra P, Udayashankar N K (2012). J Porous Mater.

[26] Whitby M, Cagnon L, Thanou M, Quirke N (2008). Nano Lett.

[27] Mattia D, Leese H, Lee K P (2015). J Membr Sci.

[28] Petukhov D I, Napolskii K S, Berekchiyan M V, Lebedev A G, Eliseev A A (2013). ACS Appl Mater Interfaces.

