# Supplementary material for: Liquid permeation and chemical stability of anodic alumina membranes
Source: Beilstein J Nanotechnol. 2017 Mar 6;8:561–70. doi: 10.3762/bjnano.8.60 (PMC5355881; doi:10.3762/bjnano.8.60)
Supplement: File 1 — Thermal analysis and membrane modification via CVD process. [file Beilstein_J_Nanotechnol-08-561-s001.pdf]

Supporting Information

for

Liquid permeation and chemical stability of anodic alumina membranes

Dmitrii I. Petukhov[*1,2], Dmitrii A. Buldakov[2], Alexey A. Tishkin[2], Alexey V. Lukashin[1,2], Andrei A. Eliseev[1,2]

Address: [1]Department of Chemistry, Lomonosov Moscow State University, Moscow 119991 Leninskie hills 1–3, Russia and [2]Department of Materials Science, Lomonosov Moscow State University, Moscow 119991 Leninskie hills, Russia

Email: Dmitrii I. Petukhov - di.petukhov@gmail.com

[*] Corresponding author

Thermal analysis and membrane modification via CVD process

Thermal analysis

Thermal analysis of membranes obtained in mild and hard anodization conditions (with average pore diameters of 40 ± 5 and 90 ± 10 nm correspondingly) indicates the only major difference associated with a mass loss at the temperature range of 100–500 °C (Figure S1). Here we attribute this loss to the oxolation process and partial loss of coordination water from highly soluble aluminum polyhydroxocomplexes. Moreover the water loss quantity is well consistent with the fraction of octahedrally coordinated aluminum, centered at 7 ppm in NMR spectra, which is removed during water filtration (see main text for details). At higher temperatures (800–1000 °C) partial crystallization of AAO takes place, accompanied by the absorbed CO_2 evolution

from the structure. The quantity of absorbed CO_2 is similar in the membranes obtained in mild and hard anodization conditions and does not changes much after filtration of 0.5 m^3/m^2 of pure water (Figure S2). This point out a crucial role of alumina polyhydroxocomplexes rather than $C_2O_4^{2-}$ coordinated aluminum in blocking the porous structure of AAO during water permeation.

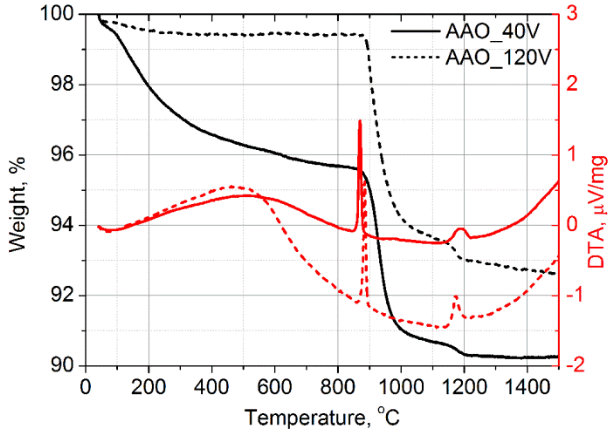

Figure S1: Thermal analysis results for membranes obtained in mild and hard anodization conditions.

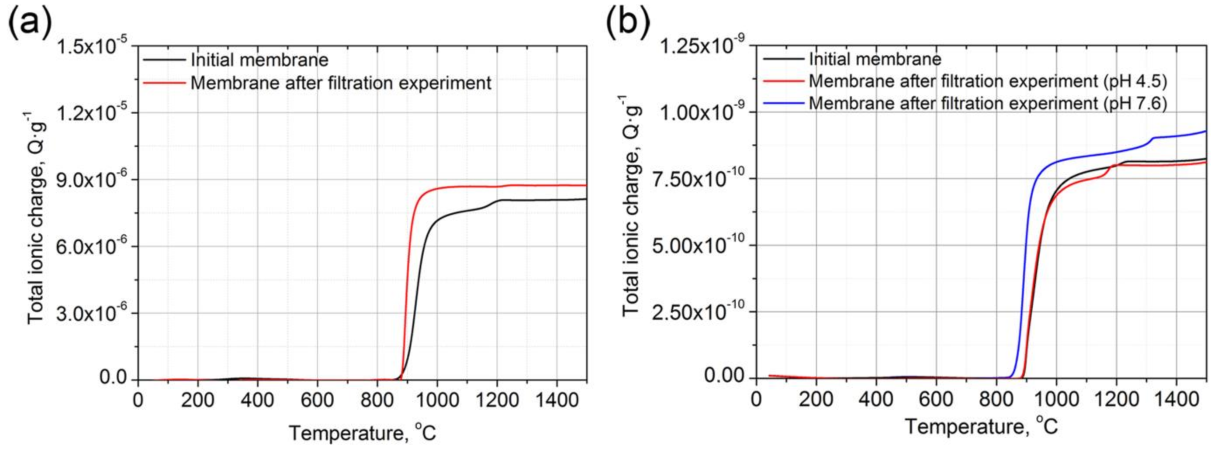

Figure S2: Integral MS-Signal for mass number 44 (CO_2) normalized to sample weight for membranes obtained in mild (a) and hard (b) anodization conditions in initial state and after filtration experiment.

Membrane modification via CVD process

Carbon layer was deposited onto anodic alumina pore walls by CVD from 15 vol % C_3H_6 in He (total gas flux equal to 150 mL/min) at 600 °C for two hours. To prevent curling or breaking of

membrane during heat treatment, heating and cooling rates were set to 1 °C/min. The phase composition analysis of formed carbon layer was performed using Raman spectroscopy (Renishaw InVia spectrometer with He-Ne excitation laser, λ = 633 nm). The obtained Raman spectrum is shown on the Figure S3. The relative intensity of the peak at about 1350 cm^{-1}, which is attribute to D-band is nearly the same to the intensity of the peak at about 1580 cm^{-1} (G-band), so we can conclude the formed carbon layer is amorphous, the same results was early published in [1].

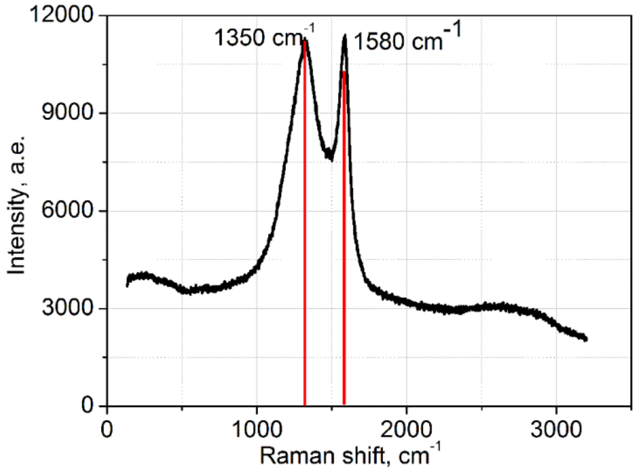

Figure S3: Raman spectrum of obtained carbon coating.

References

1. Mattia, D.; Rossi, M. P.; Kim, B. M.; Korneva, G.; Bau, H. H.; Gogotsi, Y. *J. Phys. Chem. B* **2004,** *10,* 9850–9855. doi:10.1021/jp061138s
